# Immediate Root Filling

**Published:** 1888-05

**Authors:** H. A. Smith

**Affiliations:** Cincinnati, O.


### Immediate Root Filling.

BY H. A. SMITH, D.D S., CINCINNATI, O.

[Read before the Mississippi Valley Dental Association, held at Cincinnati, O., March 8, 1888.]

If we were only called upon to fill root canals in teeth from which healthy pulps had been removed by surgical means, I presume the system of "Immediate Root Filling" would be generally adopted. Cases presenting this favorable condition are, however, comparatively rare, and the dentist finds pulps in all stages of inflammation, decomposing and dead pulps, complicated with disease of the peridental membrane and alveolar abscess, either acute or chronic. It is due to this variety in the cases presented, that practitioners necessarily differ in their methods of treatment and in what they regard as the proper time for permanently stopping a root. If there were no other reason for these differences in practice, they would naturally result from tempermental differences in dentists themselves. The late Prof. James Taylor, when speaking of this subject, would say, that we have a class of good practitioners who are forever treating a pulpless tooth ; they appear to recognize favorable conditions, but for some reason have not the courage to fill the root canal permanently, fearing possible subsequent trouble.

The typical immediate root filler of our day certainly shows no timidity in this direction; indeed, his courage seems oftener to run away with his judgment. He is ambitious not only to annihilate space, but time also.

The most favorable cases are those in which the pulp has been removed by surgical means, leaving at the apex of the root simply an incised wound, which will heal by first intention; and in this condition, if proper aseptic precautions have been observed in the removal of the pulp, we may fill the canal immediately, with little or no danger of irritation occuring in the apical space. When arsenious acid has been used and the irritant effects of the arsenic have been confined to the pulp, as shown usually by the slight pain attending its removal, we may also, with comparative safety, proceed to fill at once. If, however, in taking away the pulp any considerable hemorrhage follows, that is not arrested spontaneously, it is not best to fill at the same sitting. The application of carbolic acid or the usual styptics to hasten coagulation, will cause more or less injury to the tissues about the apex, inducing irritation. The better practice in such cases is to leave the clot that has been formed in contact with the wound until healing has commenced. The blood clot is nature's own dressing and completely filling, as it does, the root canal, effectually prevents infection from without. By placing an antiseptic cotton dressing in the canal, in contact with the clot, decomposition of the blood will be prevented for several days. In from twenty-four to thirty-six hours, the clot may be removed without disturbing the layer of granular tissue, which has formed, and since an antiseptic condition of the root has been maintained, we may, after careful washing proceed to fill permanently. Ordinarily in chronic cases of alveolar abscess with fistula, where systemic conditions are favorable, the root may be filled permanently, after thorough cleansing of the pulp cavity and sinus with peroxide of hydrogen, followed by a mild antiseptic such as a dilute solution of carbolic acid or bichloride of mercury.

It is in the management of those cases in which the pulp having been gangrenous, and as a source of infection for a long period has caused abscess, with discharge either through the root canal or alveolus, that a radical difference in practice prevails between the conservative practitioner and the advocate of immediate root filling. The statement that all roots can be made aseptic by treatment at one sitting we cannot accept. We have all seen how readily the dentine and cementum of roots of teeth take up coloring matter from the old-fashioned copper amalgam with which they have been filled. If this coloring matter will thus penetrate these dense tissues, certainly septic matter from a pulp that has long been gangrenous will thoroughly infiltrate the tissues of the root, often reaching the peridental membrane. We can then hope to counteract sepsis in these cases only by repeated washings with disinfectants, followed each time with an antiseptic which will not combine with organic matter in such a way as to prevent the effects of subsequent treatment. When we have decided to fill the root, the use af a powerful coagulator, such as chloride of zinc, as a last dressing is indicated. This "cooks" or "vulcanizes" the albuminous matter and renders it, as it were, fixed material. When oxychloride of zinc is used to fill the root, this effect is produced by the zinc chloride in the material.

The attempt to obtain an aseptic condition by burring out the root canal in which the pulp has been in a putrid condition for sometime, would it appears be idle; as would also the practice of correcting sepsis by introducing into the root canal the point of a heated instrument.

Those who advocate immediate root filling it appears place great reliance upon what is termed, "vis medicatrix natura." This recuperative vitality is, in the mouth, as in all parts of the body, an unknown factor, and cannot be measured accurately. Since the source of contagion in these chronic cases is not confined to the pulp chamber, we would do well to keep the root canals open until we have some better evidence of cure than the cessation of discharge through the external opening, which may follow a single treatment. Granulation tissue may form in the apical space, and along the walls of the sinus and the fistula may heal; yet a cure is not certainly effected. The balance between health and disease which obtained for a short time, has turned on the side of disease; the new tissue formation gives way and we still have a chronic case of abscess. May we not treat such an abscess through the opening in the gum after having filled the root? We may; but not nearly so effectually as when the opening is maintained through the root.

In cases of blind abscess and other trouble following this hasty method of treatment, the surgical procedure of drilling down upon the abscess is freely spoken of as an effective and easy operation. In all surgical treatment two people are to be considered, the operator and the one operated upon. The immediate root filler in the zeal of his universal method, seems to have left out of consideration the person upon whom he inflicts the wound. Besides the uncertainty of affording relief by drilling through the alveolus in cases of forming abscess, is the disagreeable fact that the operation should be performed at the most painful stage in the inflammation, that is just before the formation of pus.

The papers and discussions which have appeared on this subject in the last two years have, at least, had a wholesome effect upon our daily practice. It has resulted in the adoption of better methods of antisepsis and a stricter cleanliness of both the operator's instruments and person. The action of certain medicaments has become better known, and the relative potency of a great number of antiseptics carefully studied. More exact methods of root filling are being practiced, and we shall, no doubt, in the future have more uniformly good results in our efforts to save pulpless teeth.

